# Addressing Binocular Diplopia in a Diabetic Patient: Efficacy of Corticosteroids

**DOI:** 10.7759/cureus.65306

**Published:** 2024-07-24

**Authors:** Yulia Shtanko, Maria Stevens

**Affiliations:** 1 Humanities, Health, and Society, Florida International University, Herbert Wertheim College of Medicine, Miami, USA

**Keywords:** opthalmology ocular pathology, socioeconomic barriers to care, socioeconomic determinants, corticosteroid treatment, diabetes type 2, binocular diplopia

## Abstract

This report outlines an unusual instance involving a 65-year-old man who has a medical history of beta thalassemia minor, diabetes mellitus complicated by retinopathy, and iron deficiency anemia. He presented with binocular diplopia, a condition that frequently presents treatment difficulties. Despite inconclusive magnetic resonance imaging (MRI) findings, the patient's symptoms improved significantly with prednisone treatment. The etiology likely includes underlying retinopathy and cranial nerve palsy associated with diabetic complications. This case highlights utilizing corticosteroids, a largely unexplored treatment option, to optimize outcomes in managing binocular diplopia potentially linked to diabetic etiologies. It is notable that this patient was treated in a clinic for uninsured patients, emphasizing his low socioeconomic background, which adds further layers of complexity to his medical care. The complexities of managing diplopia in diabetes, compounded by limited treatment efficacy and financial constraints hindering complete diagnostic evaluation, underscore the intricate challenges of addressing diverse binocular diplopia presentations.

## Introduction

Diplopia, or double vision, is a complex visual symptom that can arise from a multitude of causes, both neurological and ophthalmic in origin [1]. Binocular diplopia, characterized by misaligned visual axes, often stems from neurological conditions affecting the cranial nerves responsible for eye movements. Conversely, monocular diplopia typically arises from intraocular pathology. The pathophysiology behind binocular diplopia occurs when an image does not align with the fovea in one eye, leading to the brain perceiving two distinct images. Horizontal misalignment of the eyes results in horizontal diplopia, while vertical misalignment leads to vertical diplopia [2].

There are a diverse range of etiologies that cause diplopia. The direction of diplopia being horizontal, vertical, or oblique often provides critical clues to its underlying etiology [3]. For instance, horizontal diplopia frequently results from sixth cranial nerve palsy or internuclear ophthalmoplegia, among other causes. Notably, horizontal diplopia induced by prolonged near vision can indicate convergence insufficiency, which is particularly prevalent in Parkinson's disease patients. Other, nonspecific etiologies of diplopia include brainstem lesions, lesions of the medial longitudinal fasciculus, lesions affecting the cavernous sinus, lesions of the superior orbital fissures, orbital disease seen in thyroid eye disease, disorders of neuromuscular junctions, Guillain-Barré syndrome, and thiamine deficiency (Wernicke encephalopathy) [2].

Understanding the various causative factors of diplopia is crucial, especially in individuals with diabetes. Diabetes can result in neuropathy affecting cranial nerves, particularly the third, fourth, and sixth cranial nerves [4]. Diabetic neuropathy, though relatively common, might be overlooked during diplopia investigations, despite its potential to affect these cranial nerves. It is notable that diabetes serves as the root cause in 25%-30% of individuals aged 45 years and above who experience sudden extraocular muscle palsy [5].

The epidemiology of diplopia suggests that it is common [6]. This could be due to its wide range and number of etiologies. While diplopia itself is not uncommon, binocular diplopia resulting from bilateral mononeuropathy in diabetic patients is a relatively rare complication of diabetes and poses diagnostic challenges [3].

The treatment of diplopia is not well established. While determining the underlying pathology and a thorough work-up is important, most cases resolve without treatment within several months [4]. Management of binocular diplopia include prescription glasses with prisms, eye patch, or injection of botulism toxin [2].

Given the wide array of possible etiologies and treatment challenges of binocular diplopia secondary to diabetes, this case report underscores the potential treatment for individuals with diabetes presenting with diplopia, as the effectiveness of steroid use is not yet well established [1,4]. Improved understanding and recognition of such cases can significantly impact treatment strategies and enhance patient outcomes.

## Case presentation

**Figure 1:**
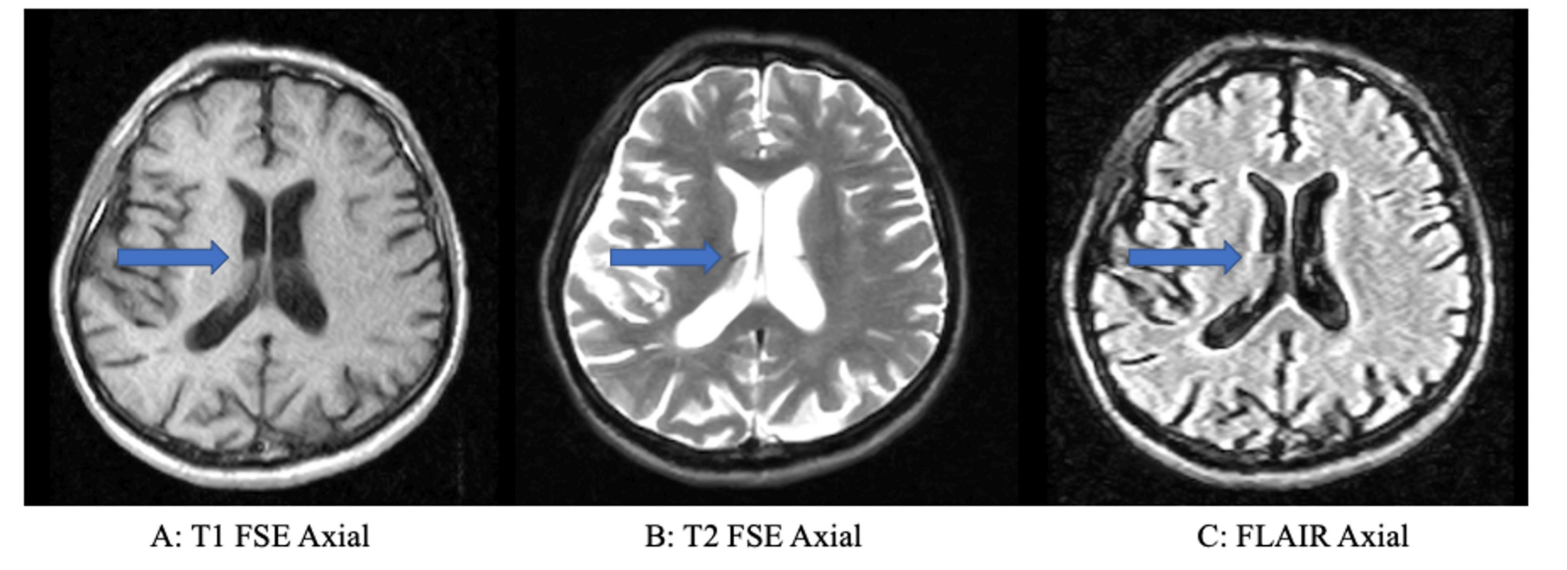
MRI of the lateral ventricles MRI: Magnetic resonance imaging; FSE: fast spin-echo; FLAIR: fluid-attenuated inversion recovery This figure depicts the MRI of the patient's lateral ventricles, indicated by the arrows. There was sinus disease and involutional changes with increased size of the lateral ventricles. A shows a T1 FSE axial view, B shows a T2 FSE axial view, and C shows the lateral ventricles in a FLAIR axial view

A 65-year-old male with a past medical history of beta thalassemia, diabetes mellitus complicated by retinopathy, and iron deficiency anemia experienced sudden-onset binocular diplopia. This patient, residing in a low socioeconomic background and lacking insurance coverage, sought medical attention at a free, community-based clinic for his symptoms. This limited the overall progression of care and proper referrals due to financial restraints. However, after 10 days of symptoms, he was seen by an ophthalmologist who recommended an eye patch and magnetic resonance imaging (MRI) study of the brain. The MRI demonstrated sinus disease and involutional changes with increased size of lateral ventricles (Figure 1).

**Figure 2:**
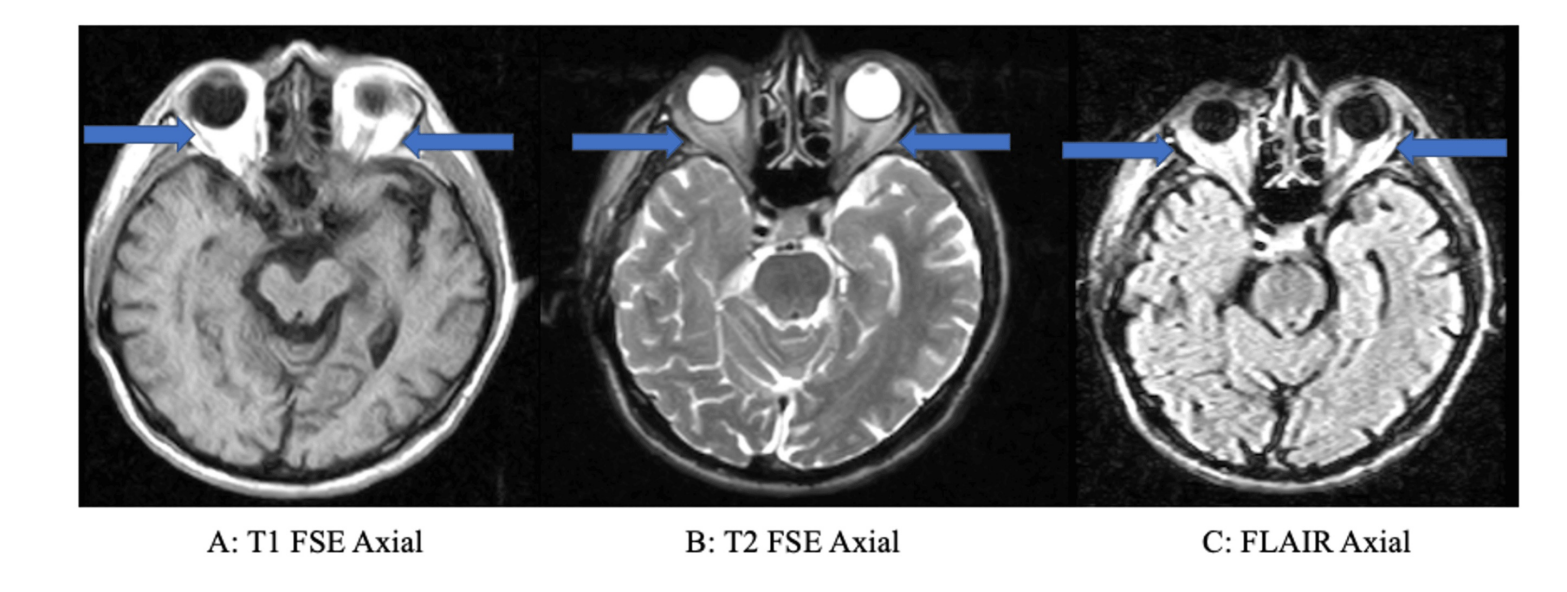
MRI of orbitals MRI: Magnetic resonance imaging; FSE: fast spin-echo; FLAIR: fluid-attenuated inversion recovery This figure demonstrates the orbits, depicted by the blue arrows. The orbits were noted to be grossly unremarkable; however, the radiologist noted that the MRI study was limited due to the signal-to-noise ratio. A depicts the brain MRI and orbits in T1 FSE axial view, B does so in T2 FSE axial view, and C does so with FLAIR axial view

There was no evidence of acute hemorrhage or masses. The orbits were noted to be grossly unremarkable; however, the radiologist noted that the MRI study was limited due to the signal-to-noise ratio (Figure 2). 

The patient did not report a trigger or inciting event. Due to the diplopia, his gait was impaired, and he had increased unsteadiness. He noted increased forgetfulness, but he was otherwise functioning at his baseline. He denied any falls or other trauma, he denied difficulty hearing, eye dryness or irritation, headache, facial numbness, tingling, pain, weakness, or slurred speech. The symptoms were constant, and no actions exacerbated them; however, head tilting improved his symptoms moderately. On physical exam, his cranial nerves were grossly intact at the time of the visit, sensation was grossly intact, finger to nose test showed no tremor, and there was a negative Romberg sign. Grossly, there was no ptosis, misalignment, horizontal or vertical movements, or deviation of his eyes that was seen on physical exam at the time of diagnosis. 

His symptoms did not improve after two months of utilizing the eye patch, as recommended by ophthalmology, and further work-up was initiated. C-reactive protein, sedimentation rate, and antinuclear antibody (ANA) was analyzed (Table 1). The normal laboratory results decreased suspicion of an inflammatory or autoimmune process resulting in his symptoms.

**Table 1:** Laboratory results ANA: Antinuclear antibody; Ab: antibody; Sed rate: sedimentation rate; TSH: thyroid-stimulating hormone This table describes the patient's laboratory results. His C-reactive protein and sedimentation rate were within normal limits. His ANA was negative. His TSH was also normal

Test	Result
C-reactive protein	0.3 mg/L (0.3-1.0mg/L)
Sed rate by modified Westergren	6 mm/h (0-20mm/h)
ANA multiplex w/reflex 11 Ab cascade	Negative
Thyroid-stimulating hormone (TSH)	2.35 mlU/L (0.5 to 5.0 mIU/L)

Due to the lack of improvement with the eye patch for several months, prednisone 20 mg, three tablets for five days, then two tablets for three days, and then one tablet for one day, were initiated. At a follow-up appointment after completing the prednisone treatment, his symptoms markedly improved but were not fully resolved. He was therefore started on another round of prednisone. He was additionally examined by an optometrist and diagnosed with moderate nonproliferative retinopathy and esotropia. It is probable that the esotropia stemmed from cranial nerve palsy, a condition likely linked to the retinopathy. Further work-up was recommended to test for the need of prism lenses, but the patient was unable to do so due to financial constraints. 

After another month, the follow-up exam demonstrated further improvement but not complete resolution of symptoms. Another prednisone course was started, after which his symptoms completely resolved. 

Before initiating steroid treatment, his diabetes was moderately controlled with a hemoglobin A1C of 7.3 on metformin 850 mg twice a day and glimepiride 2 mg with meals. He has had diabetes since his thirties with varying degrees of control throughout his life. After completion of the steroid treatments, he went to an outside clinic were his blood glucose was elevated. Therefore, his regimen was adjusted to include insulin glargine 100 unit/mL subcutaneously, five units once a day, glimepiride 2 mg once a day, and metformin 1,000 mg twice a day. He remained on this regimen with improved glucose control based on home monitoring. 

The patient was treated in an outside country for a similar episode of blurry vision and diplopia six months ago, and therefore, records were not obtainable. According to the patient, to treat his first episode, he was given a short course of an unknown medication that improved his symptoms within two weeks, and the medication was continued by a neurologist for about two months until the symptoms completely resolved. According to the patient, they performed an MRI that showed decreased blood flow to one of the cranial nerves. His second episode seems to be similar in nature with the most likely etiology being microvascular cranial nerve palsy.

## Discussion

Diabetic neuropathy, often involving the third, fourth, or sixth cranial nerves, presents a significant cause of extraocular motility disorders, especially in patients older than 45 years [4,5]. This can manifest as binocular diplopia, which is not often seen in diabetic patients. Though typically resolving within three months, recurrent episodes and involvement of multiple cranial nerves can occur. Generally, treatment involves watchful waiting, and the use of medication has not been thoroughly investigated.

Other case reports indicate various etiologies of diplopia. For instance, a 41-year-old woman had isolated horizontal binocular diplopia attributed to a lateral cavernous sinus meningioma [7]. Additionally, viral infections, such as the Omicron variant of COVID-19, have been implicated in causing oculomotor nerve palsies [8]. This emphasizes the need for comprehensive diagnostic approaches.

While most cases resolve on their own within several months, treatment with steroids has been shown to be efficacious in case studies. The case of oculomotor nerve palsy due to COVID-19 was resolved with steroid treatment. A case of idiopathic unilateral cranial nerve III palsy in a healthy 75-year-old man responded well to steroid treatment [9]. Moreover, bilateral extraocular myositis, a condition observed in individuals with type 2 diabetes, responded well to steroids [10]. Steroid therapy has also shown promise in various ophthalmologic conditions, such as optic neuritis, Graves' ophthalmopathy, and ocular myasthenia gravis, illustrating its potential benefit in managing diplopia [11-13]. Specifically, moderate dose daily prednisone for four to six weeks was shown to control diplopia in patients with ocular myasthenia gravis [13]. However, its role in the treatment of binocular diplopia, especially in diabetic mononeuropathies or unresolving cases, has not been fully explored. Overall, while management of diplopia remains uncertain, steroid use should be further explored due to demonstrated efficacy in case studies. 

This case highlights a 65-year-old male who developed binocular diplopia, likely secondary to a 30-year history of diabetes mellitus complicated by diabetic retinopathy. His symptoms did not improve with conservative management after two months. However, after three courses of steroids over three months, his symptoms completely resolved. Importantly, this patient’s socioeconomic limitations made his access to healthcare limited. Other patients in similar situations may not afford an extensive work-up for symptoms. Likewise, they may struggle to maintain employment or fulfill familial obligations due to their symptoms, underscoring the importance of timely treatment. This case report serves as a potential example of utilizing corticosteroids for unresolving binocular diplopia, likely due to diabetic-associated neuropathy of the cranial nerves.

## Conclusions

Diplopia serves as a common presenting feature in both neurological and ocular conditions. This case highlighted how a 65-year-old male with a past medical history of diabetes mellitus presenting with recurrent binocular diplopia responded positively to corticosteroids; however, additional research is needed to understand their exact role in managing diplopia associated with different pathologies, particularly in diabetic neuropathies and cases resistant to spontaneous recovery. Furthermore, the socioeconomic factors exacerbating the patient's condition underscore the importance of addressing disparities in access to healthcare and the need for tailored interventions in similar cases.
